# Effects of *Atractylodes Macrocephala Rhizoma* polysaccharide on intestinal microbiota composition in rats with mammary gland hyperplasia

**DOI:** 10.3389/fendo.2022.1102605

**Published:** 2023-01-25

**Authors:** Yang Ping, Changxu Li, Lihong Wang, Hong Zhao

**Affiliations:** College of Pharmacy, Jiamusi University, Jiamusi, Heilongjiang, China

**Keywords:** *Atractylodes Macrocephala Rhizoma* polysaccharide, mammary gland hyperplasia, intestinal microbial, endocrine disorders, high-throughput sequencing

## Abstract

**Background:**

In recent years, mammary gland hyperplasia (MGH) has been considered to be one of the diseases caused by endocrine disorders. It has been shown that diseases caused by endocrine disorders can be treated by regulating intestinal microbial. As a commonly used medicine in clinical practice, *Atractylodes Macrocephala Rhizoma* has good functions in regulating intestinal homeostasis. Therefore, this paper studied the effect of *Atractylodes Macrocephala Rhizoma* polysaccharide (AMP) on the intestinal flora of MGH rats, providing a new idea for polysaccharide treatment of MGH.

**Materials and methods:**

Eighteen female SD rats were selected and randomly divided into three groups: blank control group (Con), model control group (Mod), and AMP group, six rats in each group. MGH rat models were established by estradiol-progesterone combination and treated with AMP gastric infusion. The levels of E_2_, P, and PRL in the serum of rats were measured, the intestinal contents were collected, and 16s rRNA high- throughput sequencing technology was analyzed the changes of intestinal flora in the MGH rats.

**Results:**

AMP has good therapeutic effects on MGH rats, decreasing estradiol (E_2_) and prolactin (PRL) levels and increasing progesterone (P) levels; at the same time, it can regulate the abundance and diversity of intestinal flora of MGH rats, improve the disorder of intestinal flora caused by MGH, and change the community structure, increase the abundance of beneficial flora, and decrease the abundance of pathogenic flora.

**Conclusion:**

AMP can improve the intestinal microbiological environment of MGH rats, maintain the microecological balance of intestinal microbial, and improve MGH symptoms.

## 1 Introduction

The accelerated pace of life and the recent increase in environmental pollution, unreasonable dietary habits, poor living habits, and mental factors often accompany modern women, causing a series of adverse reactions such as Mammary gland hyperplasia (MGH). MGH is a degenerative disease and progressive connective tissue growth caused by hyperplasia of mammary fiber and epithelial tissue (1). Modem medical research has shown that the development of MGH is closely linked to endocrine disorders. An imbalance in the ratio of estradiol to progesterone leads to excessive proliferation and regression of the breast parenchyma (2–4). It was reported that MGH was the disease with the highest incidence among female breast diseases, and its incidence rate is as high as 75%, and the incidence rate is still rising year by year. The age of onset is also getting lower and lower, and even some breast hyperplasia will have canceration, which has become a major health problem for women around the world (5–9). At present, for the examination and treatment of MGH, pathological biopsy or surgical resection of suspicious nodules revealed by some imaging examinations were typically utilized. Oral drugs mainly include oral sex hormone drugs (such as bromocriptine, tamoxifen, danazol, etc.) and non-sex hormone drugs (thyroxine, iodine, evening primrose oil, etc.). However, surgical resection has a large wound surface, which brings irreversible psychological and physiological damage to patients; drugs easily cause multiple toxic side effects such as menstrual blood loss, menstrual disorder, dizziness, nausea, vomiting, etc., and some drugs can also cause certain damage to the human gastrointestinal tract and central nervous system (10–12). Therefore, the search for a noninvasive, efficient treatment of MGH drugs has become a research hotspot at home and abroad.

Studies have demonstrated that endocrine imbalance can affect the composition of the intestinal microbiota and can directly or indirectly alter bacterial physiology and independent gene expression (13–15). MGH is caused by endocrine imbalance, in which the imbalance of estrogen secretion is an important signal of MGH. The abundance of intestinal microbiota is not simply related to the level of estrogen but also can affect the level of estrogen secretion. In endocrine balance, a variety of bacteria in the intestinal microbiota is involved in estrogen metabolism. For example, intestinal bacteria with structures such as β-glucuronidase and β-glucosidase can facilitate the reabsorption of estrogen through the intestine and re-enter the hepatic and intestinal circulation, eventually reaching the target organs it regulates to act (16–18). When there is an endocrine imbalance, the intestinal microbiota is altered and the β-glucuronidase content increases, raising the level of estrogen in the mammary glands. Breast epithelial cells that are chronically exposed to high levels of estrogen and become lesions occur, leading to their excessive proliferation, causing MGH (19, 20). Therefore, it was hypothesized that the treatment of MGH can be obtained by regulating the intestinal microbiota of homeostasis and balancing the level of estrogen secretion.

**Figure 1:**
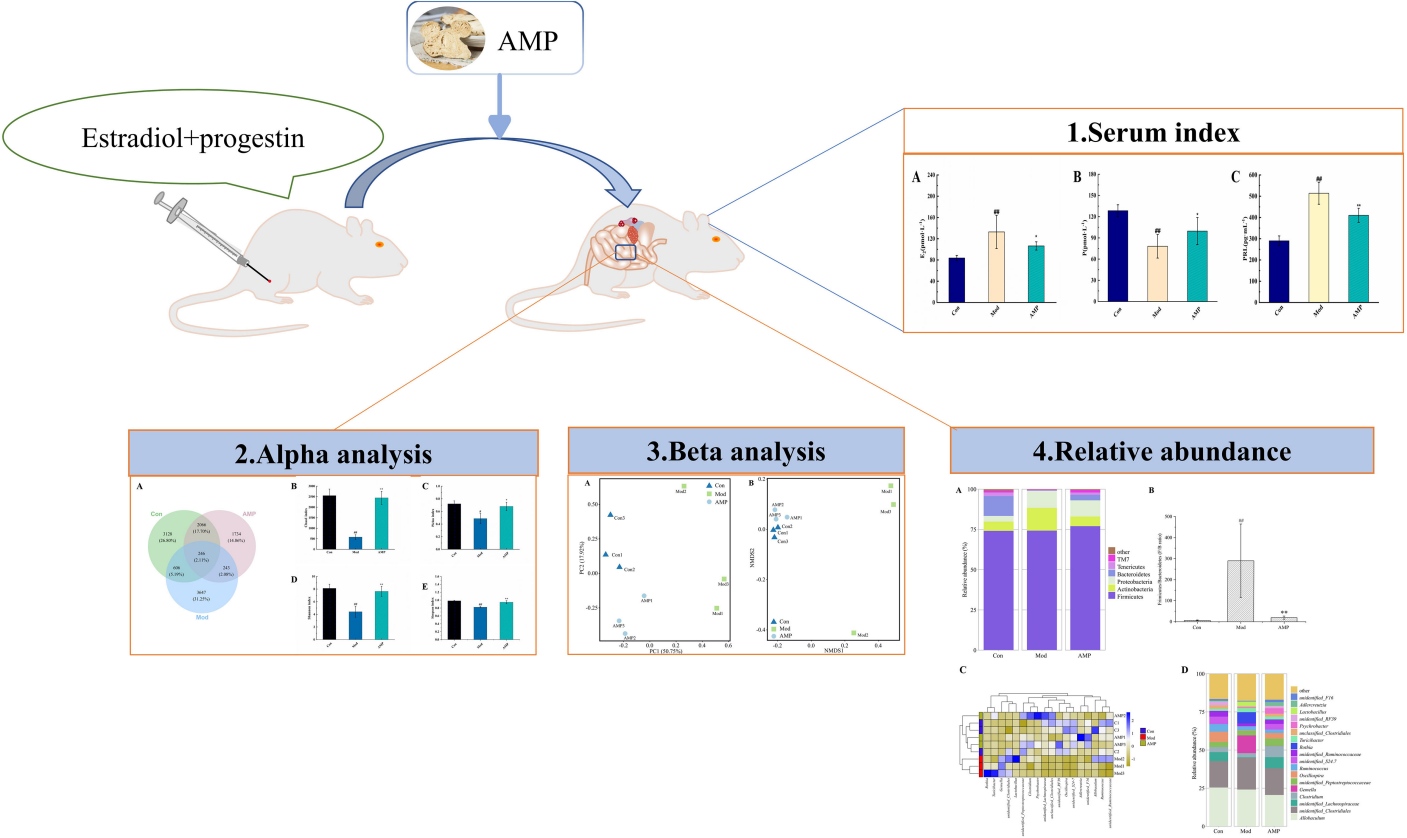
Experimental flow graph.

The earliest understanding of the mammary gland in Chinese medicine can be traced back to the Nei Jing, where it is written that “a woman’s breast belongs to the liver, while the nipple belongs to the stomach” and that “Chong and Ren meridians can regulate the Qi and blood of the 12 meridians and they stop at the breast upward and form menstruation downward”, which explains the close relationship between the breast and the spleen and stomach in terms of physiological function and meridian affiliation. Chinese herbal medicine has a very beneficial effect on the balance of the intestinal micro-ecolozy, and the intestinal microbiota coincides with the theory of “spleen and stomach” in Chinese Medicine (21–23). *Atractylodes Macrocephala Rhizoma* is the dry rhizome of the Asteraceae plant *Atractylodes macrocephala Koidz*, which has the effect of strengthening the spleen and stomach, drying and dampening water. Modern research shows that *Atractylodes Macrocephala Rhizoma* contains a variety of volatile components, polysaccharides, and amino acids, which have various pharmacological activities such as improving the function of the gastrointestinal tract and regulating the intestinal microbiota (24, 25).Therefore, this project will use AMP to treat MGH rats and analyze the effect of AMP on the intestinal microbiota of MGH rats through 16S RNA high-throughput sequencing, to provide theoretical support for the treatment of MGH from the perspective of intestinal microecology, as well as to provide an experimental basis for fully exploiting the medicinal value of AMP. The research idea is illustrated in **Figure 1**.

## 2 Materials and methods

### 2.1 Animals and reagents

Eighteen specific pathogen-free (SPF) grade female non-pregnant SD rats (200 ± 20 g) were purchased from Changchun Yisi Experimental Animal Technology Co., Ltd. (License No.: SCXK (Ji)-2018-0007) and housed in the SPF grade animal room. All animals were raised in the control room with a temperature of 24 ± 2°C and a humidity of 60% ± 5%. The light–dark cycle was 12/12 h. All experimental procedures involving animals were approved by the Animal Ethics Committee of the Animal Experimental Center of Jiamusi University.

Estradiol benzoate and progesterone injection were purchased from Shanghai Quanyu Biotechnology (Zhumadian) Animal Pharmaceutical Co., Ltd. Estradiol (E_2_), progesterone (P), and prolactin (PRL) kits were purchased from Jiangsu Enzyme Immunity Industrial Co., Ltd.

### 2.2 Medicine

*Atractylodes Macrocephala Rhizoma* was purchased from Jiamusi Baicaotang pharmacy.

According to the previous research method to prepare AMP, take degreased *Atractylodes Macrocephala Rhizoma*, add distilled water according to the ratio of the material to liquid of 1:18 g · ml^-1^; reflux at 90°C for extraction for 3 h, three times; combine the filtrates; concentrate under reduced pressure; centrifuge; add absolute ethanol to the filtrate, with supernatant ethanol concentration of 80%; stand at 4°C for 12 h; centrifuge; collect precipitation; redissolve; concentrate; freeze dry; and then obtain the *Atractylodes Macrocephala Rhizoma* crude polysaccharide. AMP with a polysaccharide content of 64.23% was obtained after deproteinization using the Sevage method.

### 2.3 Experimental design

Eighteen female SD rats were chosen and fed adaptively for 7 days. They were randomly divided into the blank control group (Con), model control group (Mod), and AMP group, with six rats in each group. Except for the Con group, which was injected with the same amount of normal saline intramuscularly, rats in the other groups were injected with estradiol benzoate 0.5 mg·kg^-1^·D^-1^ intramuscularly for 25 days, followed by progesterone 5 mg·kg^-1^·D^-1^ intramuscularly for 5 days. After successful modeling, rats in the AMP group were given a 280 mg·kg^-1^·D^-1^ polysaccharide solution, and rats in Con and Mod groups were given the same amount of normal saline for 30 days.

#### 2.3.1 Collection and detection of serum samples

After the last administration, rats in each group fasted for 12h. After weighing, rats were anesthetized by intraperitoneal injection of 2% pentobarbital sodium. The rats were done by cervical dislocation. Blood was drawn from the abdominal aorta and placed in the procoagulant tube for 20 min to separate the serum. The serum levels of E_2_, P, and PRL were identified by ELISA.

#### 2.3.2 Collection of intestinal cecal content samples

After taking blood from the abdominal aorta, the rats were quickly executed by cervical dislocation. Under aseptic conditions, the cecal content was taken with sterilized forceps and placed in a 5 ml sterile EP tube, numbered, weighed, and quickly placed in liquid nitrogen. After the sample is collected, it was moved to -80°C for storage.

#### 2.3.3 16S rRNA genes high-throughput sequencing

Take the cecal content. The total DNA in content was extracted in strict accordance with the DNA extraction kit. A nanodrop spectrophotometer was utilized to quantify DNA (DNA quantitative analysis), and the purity and concentration of the extracted genomic DNA were identified by electrophoresis. Amplification was performed using the 16S rDNA V3-V4 variable region, and the Quant-it PicoGreen dsDNA Assay Kit was used for fluorescence quantification. For further fluorescence quantification, samples should be combined in the appropriate proportions. Afterward, Illumina’s NovaSeq 6000 sequencer was utilized for double-end sequencing of rat cecal contents.

#### 2.3.4 Bioinformatics and statistical analysis

OTU clustering of non-repetitive sequences was made with 97% similarity using QIIME2 DADA2 software. Species classification was annotated using the Greensenes database (release 13.8). alpha-Diversity indices (Chao1, ACE, Simpson, and Shannon indices) were calculated to obtain the species richness and diversity. Meanwhile, the beta-diversity was analyzed in the gut microbiota of different samples; the similarity and difference of community composition between samples (or subpopulations) were obtained by analyzing the beta-diversity of gut microbiota in different samples.

SPSS 26.0 statistical software was used for data analysis. All data in the experiment were expressed as mean ± standard deviation (X ± s). One-way ANOVA was used for comparison between groups. p < 0.05 was considered statistically significant. The drawing is expected to be completed by using the R 4.1.3 and GraphPad Prism 8 software.

## 3 Results

### 3.1 General condition of rats and the effect of AMP on the serum indicators

**Figure 2:**
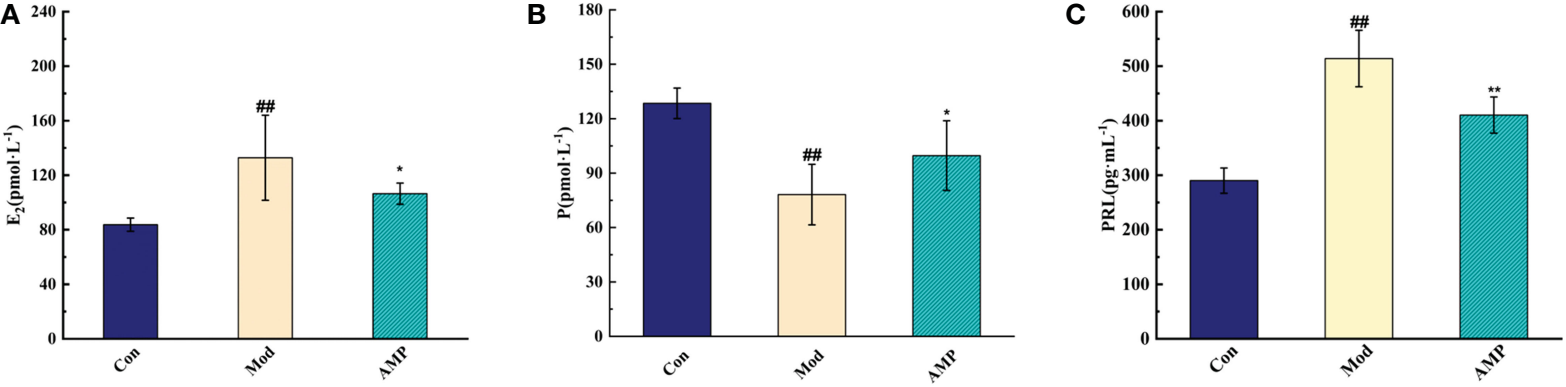
Effect of AMP on serum indexes of MGH rats, n = 6 **(A)**, effect of AMP on E_2_ of MGH rats; **(B)**, effect of AMP on P of MGH rats; **(C)**, effect of AMP on PRL of MGH rats). Compared with Con group, # represents P < 0.05, ## represents P < 0.01; Compared with Mod group, * represents P < 0.05, ** represents P < 0.01.

Before modeling, the rats had normal appetite and response, smooth hair, and dry feces. After modeling, Mod group rats had poor appetite, listlessness, and no light hair, and the body weight decreased significantly compared with the Con group. The rat teats were red, swollen, and elevated, and some rats showed thin, soft, and yellow watery stools, which proved that the MGH model was successfully prepared. After the AMP treatment intervention, the AMP group rats returned to their gradually normalized food intake, the body weight increased significantly, and the nipple swelling and diarrhea symptoms of rats were alleviated. Furthermore, it was suggested that AMP had a certain therapeutic effect on MGH model rats, and it can improve the unfavorable symptoms caused by breast hyperplasia. As showed in **Figure 2**, compared with the Con group, the serum levels of E_2_ and PRL in the Mod group rats were significantly increased (p < 0.05), and P levels were significantly decreased (p < 0.01). Compared to the Mod group, serum levels of E_2_ and PRL levels in the AMP group were significantly increased (p < 0.01), and P levels were significantly decreased (p < 0.05).

### 3.2 Sequencing data quality assessment of intestinal contents microbiota

**Figure 3:**
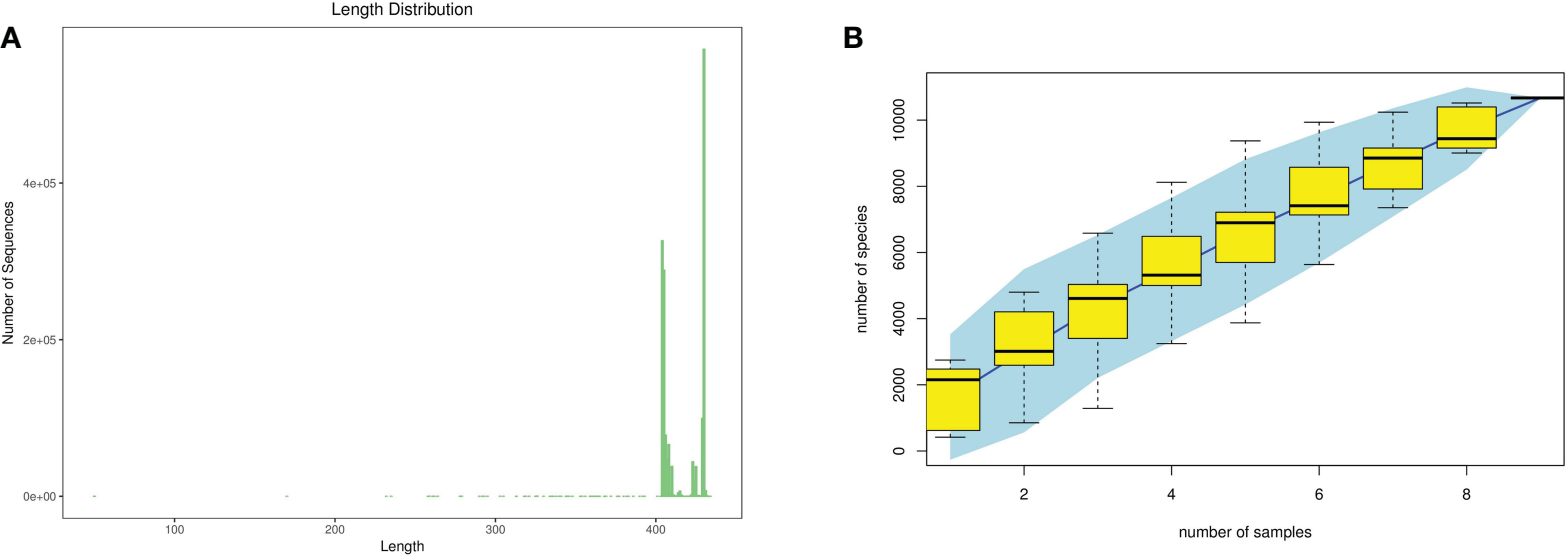
Quality evaluations of sequencing data of rat intestinal contents microbiota **(A)**, sequence length distribution diagram; **(B)**, species accumulation curve diagram).

According to the statistics of the length of the sequence obtained in this sequencing, the length of the sequence obtained in each sample is concentrated at around 400–500 bp. As shown in **Figure 3**, the samples continued to increase, the rate of increase in OTU number slowed down, and the curve tended to flatten, demonstrating that with the addition of new samples, the total number of OTUs almost did not increase, which proved that the sample of this study was sufficient to meet the needs of the study.

### 3.3 Effect of AMP on OTU number and alpha-diversity of intestinal contents in the MGH rats

**Figure 4:**
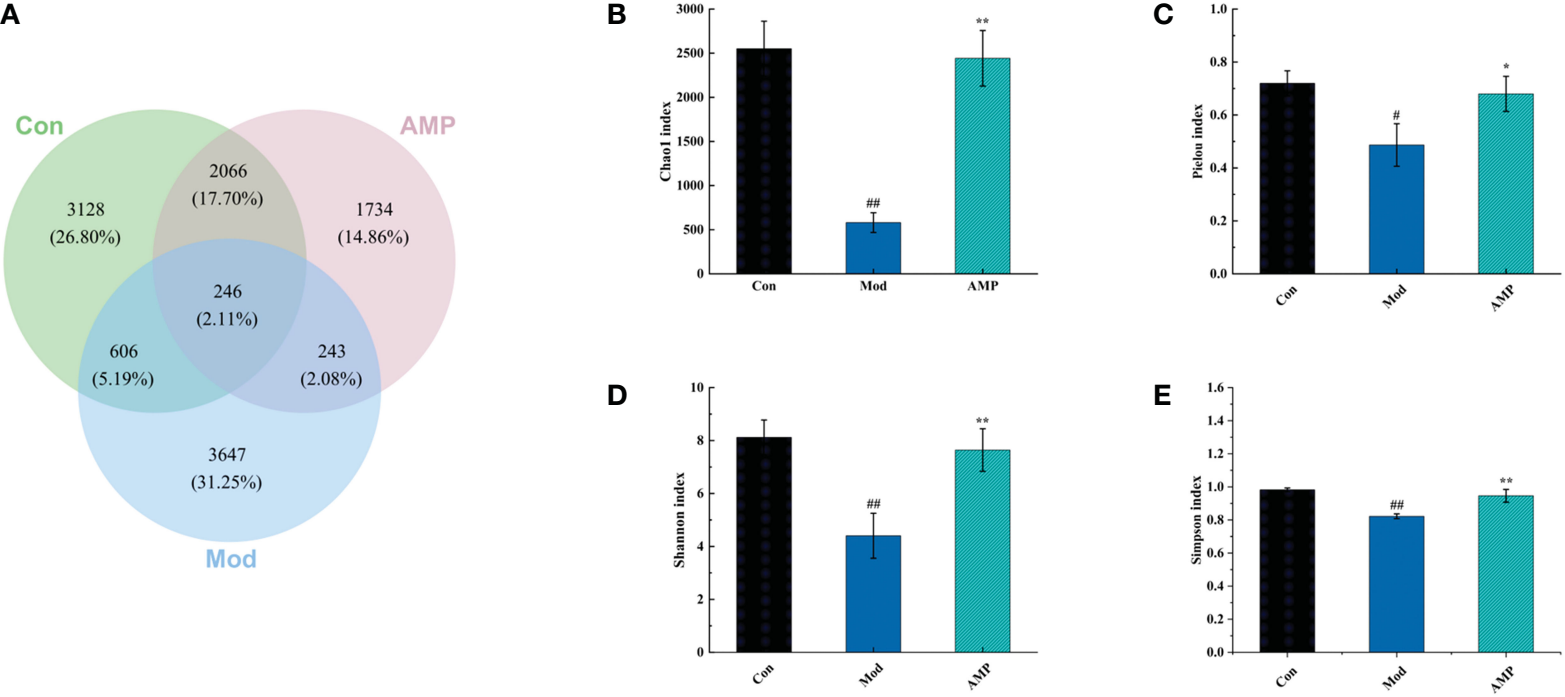
Effect of AMP on the number of intestinal OUT and alpha diversity of MGH rats **(A)**, number of intestinal OUT of rats; **(B)**, Chao1 index; **(C)**, Pielou index; **(D)**, Shannon index; **(E)**, Simpson index). Compared with Con group, # represents P < 0.05, ## represents P < 0.01; Compared with Mod group, * represents P < 0.05, ** represents P < 0.01.

OUT and Venn diagrams reflected the similarity and overlap of the bacterial community of different samples and visualized the similarity and uniqueness of sample points at the OTU level. As show in **Figure 4**, The total OTUs in the Con, Mod, and AMP groups were 3128, 3647, and 1734, respectively, and the number of OTUs in the intersection of the three groups was 246. The OTU number in the Mod group was significantly higher than the Con group. MGH disease can lead to the imbalance of intestinal microbiota in rats and increase in the number of harmful microbiota. After treatment with AMP, the OTU number in the rat intestine decreased significantly but was still lower than that in the Con group.

alpha-Diversity describes the biodiversity within a given area or ecosystem, i.e., it assesses the biodiversity of a given sample and is usually characterized by the calculation of diversity indices based on species richness or evenness, e.g., Chao1 and Pielou indices are often used to estimate the total number of species in a community: the larger the index, the greater the total number of community species. Shannon and Simpson indices can take into account both species richness and evenness, and they provide an objective picture of community species diversity. The larger the value of the Simpson index, or the smaller the value of the Shannon index, the lower the community diversity (26, 27). In the alpha analysis, the Pielou and Chao 1 indices were used to evaluate species richness, and the Simpson and Shannon index were used to evaluate microbial diversity. Compared with the Con group, the Mod group showed a highly significant decrease in Chao 1 index (p < 0.01), a significant decrease in Pielou index (p < 0.05), and Shannon index and Simpson index both highly significantly decreased (p < 0.01), indicating that MGH can reduce the richness and diversity of rat intestinal microbiota. Compared with the Mod group, the AMP group showed a highly significant increase in the Chao 1 index of rat microbiota (p < 0.01); Pielou index (p < 0.05), Shannon index (p < 0.01), and Simpson index (p < 0.01) were significantly increased, indicating that AMP could increase the abundance and diversity of intestinal microbiota in MGH rats.

### 3.4 Effect of AMP on intestinal beta-diversity in MGH rats

**Figure 5:**
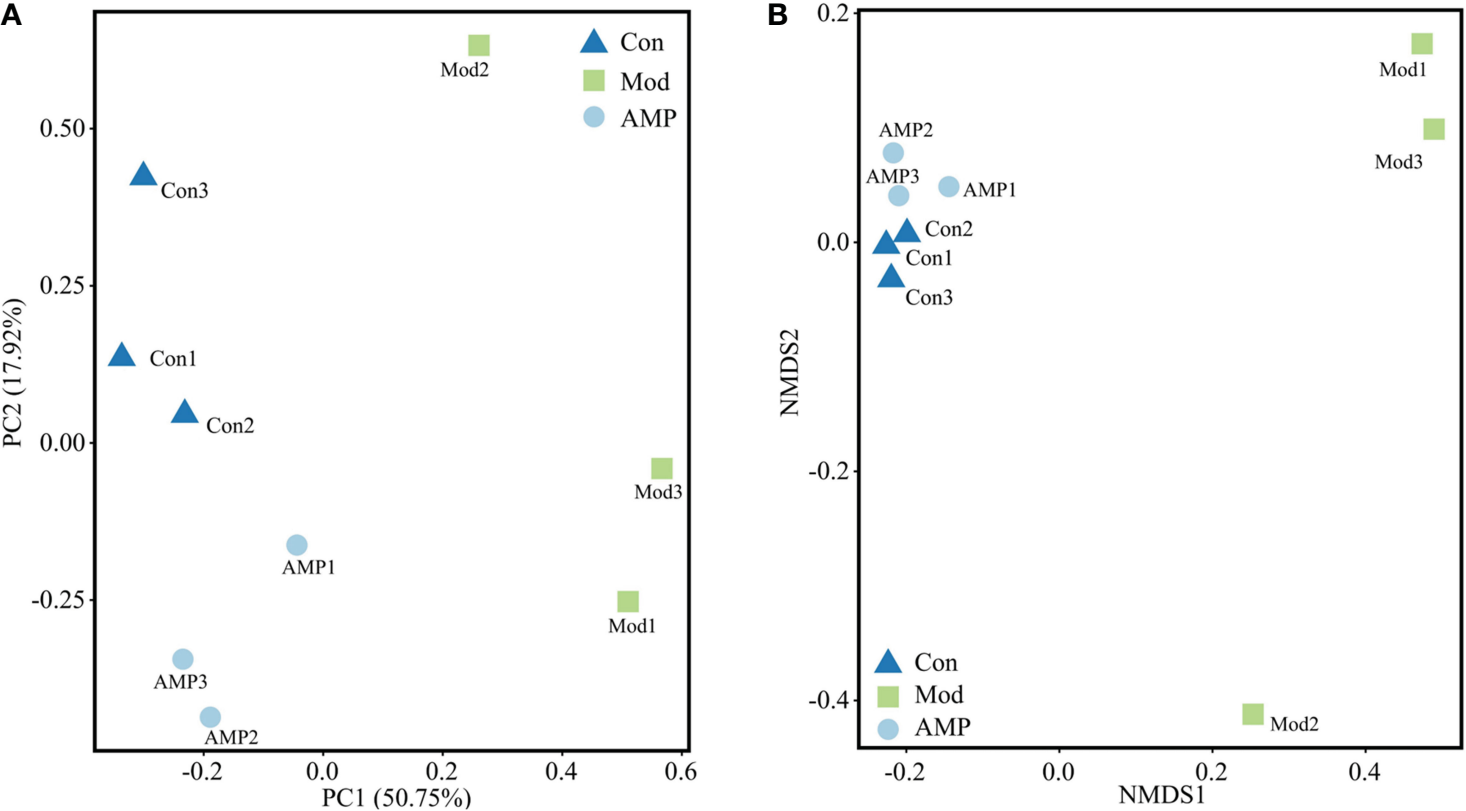
Effect of AMP on intestinal beta diversity of MGH rats **(A)**, PCA; **(B)**, NMDS).

beta-Diversity describes the differences in species composition between habitat communities, i.e., the differences between samples. Principal component analysis (PCA) is a simplified data analysis technique that can reflect differences and distances between samples by analyzing sample composition at 97% similarity. PCA uses variance decomposition to reflect differences across multiple data sets on a two-dimensional coordinate plot, with the axis distance best reflecting the two eigenvalues of the variance value. The more similar the sample composition, the closer the distances reflected in the PCA plot (28). As showed in **Figure 5A**, the principal component variable 1 was 50.75% and the principal component variable 2 was 17.92%. The distance between the Con group and Mod group was significantly different, indicating that MGH had a certain effect on the composition of intestinal microbiota in rats. Compared with the Con group, the distance between the AMP group was small and relatively concentrated. The distance between points in non-metric analysis (NMDS) reflects the difference between samples and groups. The more distance, the more different, and vice versa. As shown in **Figure 5B**, compared with the Con group, the Mod group was relatively dispersed, indicating that the species was very different. Compared with the Mod group, the AMP group was relatively concentrated, which was closer to the Con group, indicating that there was little difference in species composition within the groups. The PCA and NMDs showed that the AMP had a significant restored effect on the species composition of intestinal microbiota in MGH rats.

### 3.5 Effect of AMP on the relative abundance of intestinal microbiota in MGH rats

**Figure 6:**
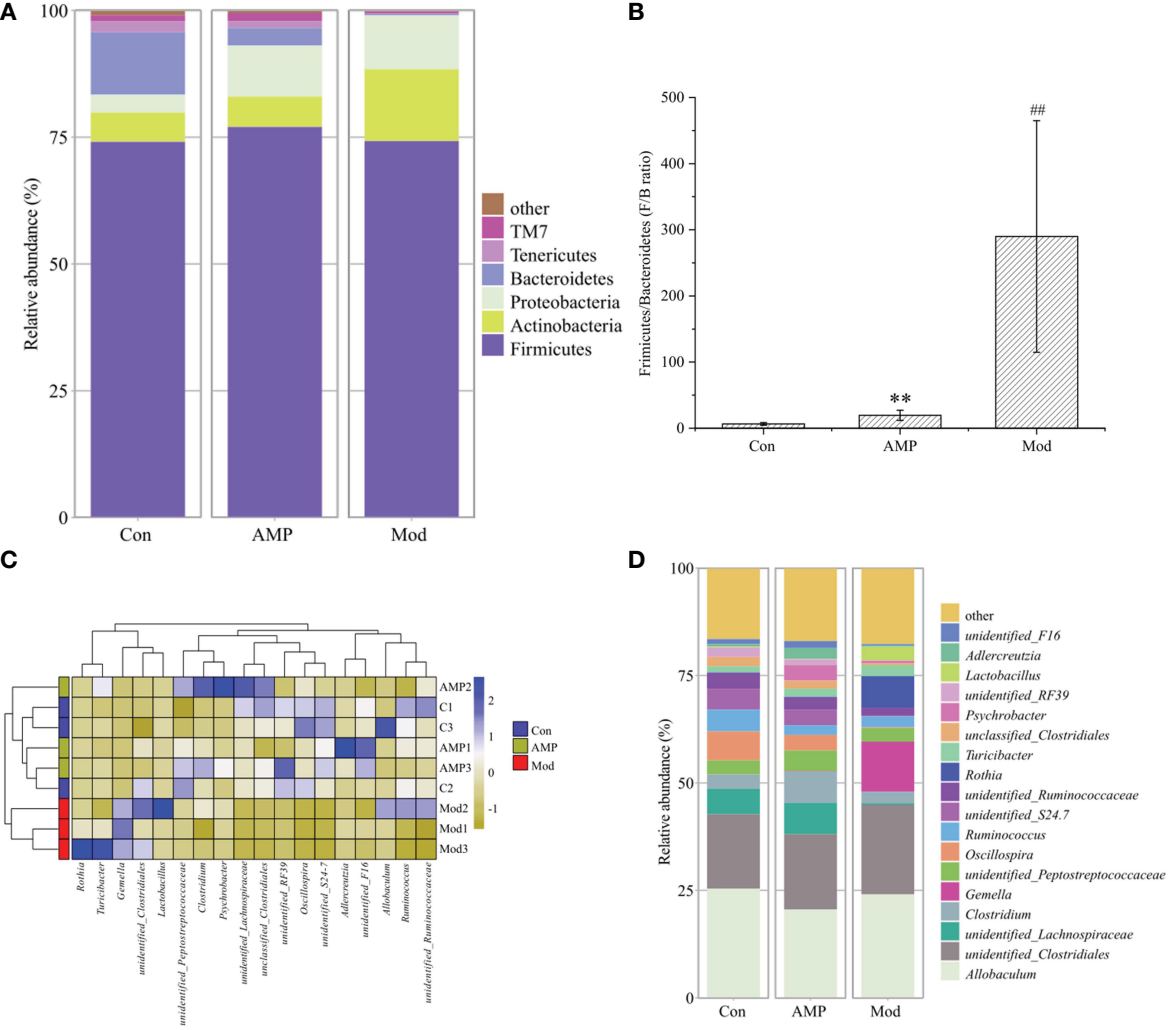
Effect of AMP on the relative abundance of intestinal microbiota in MGH rats (relative abundance **(A)**, phyla level; **(B)**, Firmicutes/Bacteroides; thermogram of relative abundance at **(C)**, phyla level; relative abundance at **(D)**, genus level). Compared with Con group, # represents P < 0.05, ## represents P < 0.01; Compared with Mod group, * represents P < 0.05, ** represents P < 0.01.

As shown in the **Figure 6A**, the relative abundance of intestinal microbiota of MGH rats was shown at the phylum level, in which four taxa, Firmicutes, Actinobacteres, Proteobacteres, and Bacteroidetes, were the dominant phylum, accounting for a larger proportion of the total microbiota, about 95%. Compared with the Con group, in the Mod group the relative abundance of Firmicutes decreased but was not significant; the Bacteroidetes decreased significantly (p < 0.05), and the Actinobacteres and Proteobacteres increased but were not significantly different. The AMP group compared with the Mod group, the relative abundance of Firmicutes and Bacteroidetes increased, but there was no significant difference, and the Actinobacteres and Proteobacteres decreased, but there was no significant difference. **Figure 6B** shows the ratio of Firmicutes and Bacteroidetes (F/B ratio). Compared with the Con group, F/B was higher in the Mod group, and the AMP group was higher too compared to Mod group. The **Figure 6C** shows that, a heat map can simultaneously provide information on community species composition and abundance and visually reflect the similarities and differences in community composition of different samples or subgroups through color changes. In the changes of detected phyla, Con group and AMP group were clustered into one category, while Mod group was more dispersed due to the influence of MGH. As shown in **Figure 6D**, at the genus level *Allobaculum*, *Clostridiales*, *Lachnospiraceae*, and *Clostridium* were the dominant genera. Compared with the Con group, the relative abundance of Actinobacteres and Clostridium in the Mod group decreased. Lachnospiraceae decreased significantly (p < 0.05) and *Clostridiales* increased. Compared with the Mod group, the AMP group *Lachnospiraceae* was higher, *Clostridium* was highly significant (p < 0.01), *Clostridiales* was higher, and *Allobaculum* was lower.

## 4 Discussion

The human intestinal microbiome is a diverse and complex ecosystem and is home to thousands of microorganisms that co-evolve with their hosts and play important roles in health and disease. The important role of intestinal microbial in health and disease has become a research focus (28–31). Intestinal microbial diversity is tightly associated with levels of estrogen and its metabolites (32–34). A reduction in intestinal microbial diversity can reduce the activity of β-glucuronidase that can reduce the circulation form of estrogen in the body and break it down into active ingredients, resulting in a large estrogen accumulation in the mammary gland, and leading to endocrine disorders (35–37). Modern medicine also believes that the occurrence of MGH is linked to endocrine disorders, in particular, it is related to the imbalance of balance hypothalamic-pituitary-ovarian-mammary gland endocrine axis, in which the secretion of estrogen increases and the secretion of progesterone decreases. The imbalance of female/progesterone ratio is the main cause of mammary gland hyperplasia (38–41). The mammary gland is an important target organ for the effect of estrogen, and when estrogen secretion in the female organism is too high, it will cause the female breast tissue to be in a state of over-stimulation, and the poor physical condition will have an impact on the mammary gland, leading to MGH (42, 43). Metabolic enzymes changed regulated by the intestinal microbiota will affect the estrogen level change, which leads to endocrine dysregulation, eventually causing MGH (36, 44). Thus, estrogen can be participated in the development of MGH through changes in the metabolic levels of intestinal microbiota.

In this experiment, E_2_, P, and PRL were significant indicators to determine whether the rats had MGH. Compared with the Con group, the Mod group E_2_ and PRL levels were increased significantly and P levels were decreased significantly, compared with the Mod group; the AMP group E_2_ and PRL levels were reduced and P levels were increased, indicating that AMP could alleviate the symptoms on rats MGH effectively. By 16S RNA amplified sequencing, in alpha-diversity analysis, the Mod group Chao1, Pielou, Shannon, and Simpson indices were decreased in the intestinal tract of rats, indicating that MGH had an inhibitory effect on the richness and diversity of intestinal microbiota in rats. Compared with the Mod group, the AMP group Chao1, Pielou, Shannon, and Simpson indexes in the intestinal tract of rats were significantly increased, which was similar to that in the Con group, indicating that AMP had a certain improvement effect on the intestinal microbiota of rats with MGH. In the beta-diversity analysis, AMP improved the chances of microbial community structure in intestinal contents of MGH rats and gradually restored it to the normal level. However, there were still discrete individual samples in the Mod group, which may be due to the inter-individual differences.

Under normal conditions, Firmicutes and Bacteroidetes are in dynamic balance in the intestinal tract and the ratio of relative abundance is seen as an important marker of intestinal microbiota disorders, and an increase in F/B value represents an endocrine disorder on the other hand (45–47). In this experiment, MGH changed the structure and composition of the microbiota in the intestinal contents of rats, and through the phylum level analysis on the changes of the microbiota relative abundance, we can further understand how AMP treats the MGH by regulating the intestinal microbiota. In the Mod group, the relative abundance of Proteobacteres and the F/B value also increased, suggesting that MGH can lead to the imbalance of the intestinal micro-ecological environment; after AMP treatment, the relative abundance of Proteobacteres decreased, and the value was similar to the Con group; the F/B value also decreased but was still higher than that of the Con group. Therefore, it can be deduced that AMP is beneficial to restore the major intestinal microbiota. Actinobacteres are a gram-positive bacterium, which can protect the host and nutrient from attack by pathogens in the intestine (48–50). In this experiment, compared with Con group, the relative abundance of Actinobacteres in the Mod group increased, indicating that the MGH caused resistance of intestinal microbiota. Compared with Mod group, the relative abundance of Actinobacteres in the AMP group was reduced, which indicated that the administration of AMP could alleviate the intestinal microbiota disorder. According to the analysis of genus level, Clostridiales is generally considered to be related to body inflammation, *Clostridium* contains 20α-hydroxy steroid dehydrogenase, which converts glucocorticoids into androgens, when *Clostridium* is unbalanced, a large number of androgens will be synthesized, which will cause intestinal microorganisms dysregulation and eventually lead to endocrine disorders and pathological lesions in women (51, 52). Combined with the results of this experiment, compared to the Con group, the relative abundance of *Allobaculum*, Clostridiales, and Lachnospiraceae decreased and the *Clostridium* increased in the Mod group; compared to the Mod group, the relative abundance of Lachnospiraceae, *Clostridium*, Clostridiales increased, and *Allobaculum* decreased in the AMP group. Further studies are needed to investigate this difference. Based on the above experiments, AMP can maintain the endocrine balance by regulating the homeostasis of intestinal microbiota, so as to affect the occurrence and progress of MGH.

In general, MGH caused by endocrine dysregulation breaks the intestinal microbiota balance in rats, and the diversity and richness of intestinal microbiota are destroyed to varying degrees. Through the study of AMP in the treatment of MGH and its effect on intestinal microbiota, this experiment proved that AMP had a certain therapeutic effect on MGH and also had a definite recovery effect on the diversity, richness, and community structure of intestinal microbiota in MGH rats. However, the therapeutic effect of a single polysaccharide component on MGH by regulating intestinal microbiota has not been excellent. Therefore, the therapeutic effect of the AMP of MGH through intestinal microbiota needs further study.

## Data availability statement

The original contributions presented in the study are publicly available. This data can be found here: https://doi.org/10.5061/dryad.gf1vhhmtc.

## Ethics statement

The animal study was reviewed and approved by the Animal Ethics Committee of the Jiamusi University.

## Author contributions

YP prepared the drafting of manuscript and interpretation of data. CL performed the experiment and analyzed in the analyzing of part of the data. LW and HZ designed the study and revised the manuscript. All authors contributed to manuscript revision, read, and approved the submitted version.
